# Methyl Mercapturate Synthesis: An Efficient, Convenient and Simple Method

**DOI:** 10.3390/molecules13102394

**Published:** 2008-10-01

**Authors:** Benoît Cossec, Frédéric Cosnier, Manuella Burgart

**Affiliations:** Institut National de Recherche et de Sécurité, Département Polluants et Santé, Rue du Morvan CS 60027, Vandoeuvre, 54519 cedex, France

**Keywords:** Mercapturic acids, Sulfa-Michael addition, Phase Transfer Catalysis, Biomarker

## Abstract

A safe and simple method for methyl *S*-arylmercapturate synthesis is described. Thirteen such compounds, to be used afterwards in metabolism studies, have been obtained with yields ranging from 71 to 99.6%. These compounds were obtained using a sulfa-Michael addition and synthesized by adding the corresponding thiophenols to a mixture composed of methyl 2-acetamidoacrylate (MAA), potassium carbonate and a phase transfer catalyst, Aliquat 336. MAA, the initial synthon, was itself isolated in quasi quantitative yield following a fully described synthesis.

## Introduction

Aromatic hydrocarbons belong to a broad family of chemical compounds which are widely used in many industrial applications, alone or in mixtures, and as solvents and starting products for chemical synthesis [1]. Along with the ever increasing awareness and knowledge about the toxicity of aromatic hydrocarbons [2,3,4], there is a growing need for assessing reliable and specific biomarkers and for the development of sensitive analytical methods dedicated to occupational exposure biomonitoring [5,6,7,8,9,10].

**Scheme 1:**
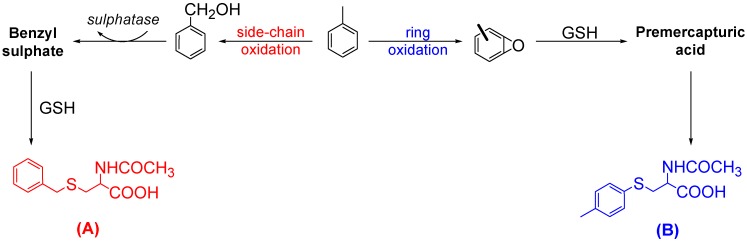


Mercapturic acids (MA), which are *N*-acetyl-l-cysteine-*S*-conjugates (or 2-acetamido-3-sulfanyl-propionic acids according to IUPAC Nomenclature) are end products of the glutathione detoxification pathway (GSH). Since Van Doorn’s preliminary studies [11], increasing attention has been paid to these minor metabolites. Over the last decades, several MA derived from aromatic hydrocarbons have been identified and their corresponding metabolic pathways have been explained [9,10,11,12,13]. In this respect, Angerer *et al*. [13,14] demonstrated that toluene and xylenes are metabolised into MA using two different oxidation pathways (Scheme 1). As an example, toluene has been shown to interact with GSH by conjugation with reactive electrophilic intermediates. The nature of these intermediates depends on the first oxidation step catalysed by cytochrome P-450 enzymes and which may occur either at the side chain or at the aromatic ring. Side chain oxidation produces benzylic alcohol, which is enzymatically transformed into a sulphate ester [11], then to a *S*-benzylmercapturic acid (**A**) once conjugated with GSH. On the other hand, aromatic ring oxidation forms an arene oxide which leads to *S*-*p*-tolylmercapturic acid (**B**) after conjugation with GSH [13].

Several research teams have concluded that MAs are more selective and sensitive than traditional biomarkers, which are generally the major metabolites [11,15,16]. During the same period, the American Conference of Govermental Industrial Hygienists (ACGIH) and the Deutsche Forschungs Gemeinschaft (DFG) decided to publish a biological exposure index for *S*-phenylmercapturic acid as a biomarker of exposure to benzene [17,18].

**Scheme 2:**
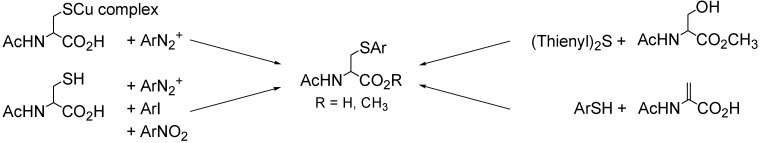


For the aforementioned reasons, a growing interest in the glutathione pathway and MA synthesis has emerged. While MAs coming from side chain oxidation are well described in the literature [19,20,21], MAs resulting from ring oxidation are still being investigated in order to improve their synthesis. Scheme 2 reports the five main procedures for *S*-arylmercapturic acids (AMA) (or ester derivative) synthesis: the reaction of aryl diazonium salts with cysteine cuprous mercaptides or with *N*-acetyl-l-cysteine [13,22,23], the copper-assisted or palladium-assisted nucleophilic substitution of aryliodides [24,25], the nucleophilic substitution of aryl nitrate or nitroarylhalide [26,27,28], a Mitsunobu-type coupling reaction [29], and finally the process of Behringer *et al*. [30,31]

Most of the processes already described gave low yields of complicated mixtures [13,22,23], or needed highly activated aromatics [28,29,31]. Moreover, they generally suffer from different drawbacks (longer reaction time, high temperature reaction, high starting material purity, rigorous dioxygen exclusion, tedious work-up) [30,31].

**Scheme 3:**
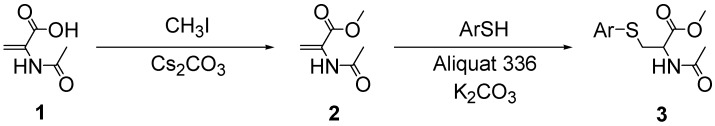


The aim of the present study was to provide gas chromatographic standards having a methyl *S*-aryl-mercapturate structure (AME) in order to use them in biological monitoring and metabolism studies. Thirteen compounds have thus been obtained in high yields from the procedure described in Scheme 3. This process is a two-step reaction starting from 2-acetamidoacrylic acid (product **1**) and involving a smooth esterification of **1** followed by a sulfa-Michael addition [32] of the adequate thiophenol on the produced acrylate (product **2,** MAA) in the presence of a phase-transfer catalyst [33].

## Results and Discussion

### Methyl 2-acetamidoacrylate synthesis (MAA; ***2***)

Few procedures have been described in the past for MAA synthesis, and conventional acidic esterification attempts to obtain this ester often failed [34,35]. This was probably due to the poor stability of the unsaturated structure. Rothstein *et al*. [34] first obtained MAA using the nucleophilic action of lead or sodium salts of compound **1** on iodomethane or dimethylsulphate, but yields never exceeded 52%. Later on, Bueno *et al*. [36], using the potassium salt of **1**, produced MAA in yields of up to 80%, but in crude form. Another conventional reagent, diazomethane, is known to be inadequate for unsaturated carboxylic acid esterification, yielding quantitatively pyrazolines structures [37]. Other procedures reported in the literature involve methyl alaninate, cysteinate or serinate in more complicated retrosynthetic mechanisms [38,39,40].

As far as we are concerned, numerous methylation and esterification methods were investigated in the laboratory [41,42], but only one reagent, cesium carbonate, was applied with success. Using experimental conditions described in the literature [43], the yield was gradually improved by increasing the ratio of iodomethane to compound **1** (Table 1). Finally, MAA was obtained quantitatively by carefully controlling the nucleophilic substitution and the time reaction.

**Table 1:** MAA (**2**) synthesis.

Entry	Cs_2_CO_3_ (equiv)	CH_3_I (equiv)	Time (h)	Yield of 2^a^ (%)
1	0.5	1.2	15	50
2	0.5	2.0	15	65
3	0.5	2.5	15	76
4	0.5	2.5	3	99.7

^a^ isolated yield

### Methyl S-p-toluylmercapturate (***3a***) synthesis: optimisation

Toluene was the first aromatic hydrocarbon to be examined when our biomonitoring studies began. Optimisation (Scheme 2, step 2) was therefore applied to obtain **3a**, a mercapturate stemming from toluene ring oxidation (Scheme 1) [13]. The results concerning this particular optimisation are summarized in Table 2. Tetrabutylammonium hydrogensulfate (TBAHS), which generally performs successfully in all kinds of phase transfer catalysis (PTC) reactions [44,45], was initially chosen as a catalyst. Using liquid-liquid conditions (entry 1), a complete deesterification of **2** unfortunately resulted. This negative result prompted us on the one hand to suppress water and to use a solid-liquid process [46,47,48] and on another hand to replace NaOH by KOH. Three reaction-condition parameters, i.e. the organic phase, the basic anion and the catalyst used, were then successively modified to improve the yields obtained.

Three solvents were thus investigated and put through the procedure. From the results of runs 3, 4 and 5, toluene may be considered as the reference solvent, other solvents appearing less suitable. This result is not in accordance with Herriott *et al*. who suggested that the reaction rate in PTC should be directly related to solvent polarity [49]. When the results of runs 9 and 10 were analysed, the relationship between solvent and yield did not appear so simple. The results observed with THF (tetrahydrofuran), which is a polar solvent, were similar to those obtained with toluene.

The investigations then moved on to screening two different bases [50]. The results obtained in runs 2 and 3, and comparatively in runs 8 and 9, clearly demonstrate that K_2_CO_3_ is superior to KOH in terms of compound **3a** formation. Furthermore, the reactions with this latter base were by far the cleanest, producing only a very minor amounts of by-products.

Two other PTC catalysts, cetyltrimethyl ammonium bromide (CTAB) and methyltrioctyl ammonium chloride (Aliquat 336), were then successively tested to conduct the sulfa-Michael addition (entries 3, 7, 8 and 9). Aliquat 336 emerged as the most active catalyst for our process (lowest quantities, highest rate).

Finally, the reaction was performed with a excess of reagent (entry 11). In fact, it is well known that arylmercaptans are particularly sensitive to oxidation. With an excess of *p*-cresol a quasi complete conversion was observed. To confirm the catalytic effect, the reaction was then carried out using the conditions described in entry 11 and avoiding the use of the selected PTC catalyst. In contrast with the preceding results, no reaction at all was observed at room temperature over a 24-hour stirring period.

In summary, appropriate solid-liquid phase-transfer catalysis conditions have been determined to carry out the addition of p-thiophenol to MAA, THF and toluene emerging as the solvents of choice. The addition takes place rapidly by successively adding a catalytic quantity of the basic anion (K_2_CO_3_) and then the catalyst (Aliquat 336) to the reaction mixture, which yields **3a**, the desired product, quasi quantitatively.

**Table 2:** Optimisation of the reaction conditions for the addition of *p*-thiocresol to compound **2**.

Entry	*p*-Thiocresol (mmol)	Base (mmol)	PTC (mmol)	Solvent	Time (h)	%Yield for 3a
1	1	NaOH 1M/H_2_O	TBAHS (0.2)	CH_2_Cl_2_/H_2_O	48	100% of **2**
2	1	NaOH (1.4)	TBAHS (0.2)	THF	48	1
3	1	KOH (1.2)	TBAHS (0.2)	THF	18	22
4	1	KOH (1.4)	TBAHS (0.2)	Acetonitrile	18	22
5	1	KOH (1.2)	TBAHS (0.2)	Toluene	18	33
6	1	KOH (1.2)	CTAB (0.2)	Toluene	48	31
7	1	KOH (1.2)	CTAB (0.2)	THF	18	49
8	1	KOH (1.2)	Aliquat 336 (0.08)	THF	18	6
9	1	K_2_CO_3_ (0.3)	Aliquat 336 (0.08)	THF	18	63
10	1	K_2_CO_3_ (0.3)	Aliquat 336 (0.08)	Toluene	18	61
11	1.4	K_2_CO_3_ (0.3)	Aliquat 336 (0.08)	Toluene	5	99.6

Amount of **2** used: 1 mmol

### Application to the synthesis of various AMEs (***3b-m***)

Using the optimised conditions established for **3a**, the Michael addition was applied to numerous thiophenols (Table 3). As expected from previous results, thiophenols bearing *para* but also *ortho* electron donating groups reacted quickly, giving the corresponding AMEs **3b-d** in remarkable yields in less than 5 hours. Similarly, thiophenols bearing two electron donating groups afforded AMEs **3e-h** in satisfactory yields, but needed generally slightly longer reaction times.

In order to complete the knowledge of this procedure, several other thiophenols were then investigated. It was successively demonstrated that a donating group was not required (**3i**), and, that despite the electron withdrawing group presence, **3j-l** can be obtained in relatively satisfactory yields with reaction times ranging from 5 to 18 hours. In addition, and unexpectedly, a thiophenol bearing five electron withdrawing groups (**3m**) gave similar results.

Finally, our procedure is safe, simple, convenient, and compatible with numerous substrates and, by and large, produced AMEs in higher yields compared to other protocols [22,23,24,25,26,27,28,29,30,31]. Furthermore, availability and low cost of Aliquat 336 are an additional favourable factor to consider.

**Table 3:** Methyl S-arylmercapturates (AME) synthesis.

Entry	AME^a^		Solvent	Time (h)	*R*_F_^c^	Yield^d^ (%)
1	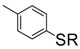	**3a**	Tol	5	0.28	99.6
2	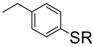	**3b**	Tol	5	0.31	99
3	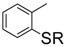	**3c**	Tol	5	0.29	94
4	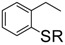	**3d**	Tol	5	0.32	96
5	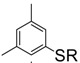	**3e**	Tol	5	0.32	95
6	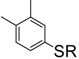	**3f**	Tol	13	0.32	90
7	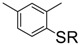	**3g**	Tol	18	0.26	82
8	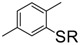	**3h**	Tol	18	0.31	98
9	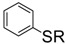	**3i**	Tol	5	0.26	96
10	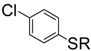	**3j**	Tol/THF^b^	18	0.27	76
11	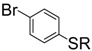	**3k**	Tol	5	0.25	88
12	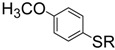	**3l**	Tol	18	0.23	76
13	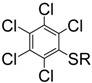	**3m**	Tol/THF^b^	18	0.26	71

^a^ R = -CH_2_-CH(NHCOCH_3_)(CO_2_CH_3_) ^b^ Tol/THF: 1/1; v/v ^c^ The reaction was monitored by thin layer chromatography using diethyl ether as eluent. ^d^ Isolated yield

## Conclusions

Methyl *S*-arylmercapturates have been synthesized according to a two-step reaction starting from compound **1** (Scheme 2) and involving a sulfa-Michael addition whose reactivity was enhanced by adding a phase transfer catalyst. A simple and efficient synthesis of MAA (**2**), the intermediary synthon, is described. The complete method proceeds smoothly with typical yields ranging from 71 to 99.6%, and may be applied to various thiophenols bearing electron donating groups or electron withdrawing groups. These compounds were then used as standards in gas chromatographic analysis and in metabolism studies.

## Experimental

### General

2-Acetamidoacrylic acid (**1**), thiophenol, chlorothiophenol, pentachlorothiophenol, 4-toluenethiol, 2-ethylthiophenol, 2-toluenethiol, cesium carbonate, tetrabutylammonium hydrogensulfate (TBAHS), cetyltrimethyl ammonium bromide (CTAB) and methyltrioctyl ammonium chloride (Aliquat 336) were purchased from Sigma-Aldrich-Fluka. 4-bromothiophenol, 2,4-dimethylthiophenol, 2,5-dimethyl-thiophenol, 3,4-dimethylthiophenol and 3,5-dimethylthiophenol were all available from Acros Organics (Fisher Bioblock, France). Finally, 4-ethylthiophenol was obtained from Avocado (Interchim, France). All the other chemicals were of analytical grade. ^1^H-NMR and ^13^C-NMR spectra were obtained on a Bruker AM 400 spectrometer operated at 400 and 100 MHz, respectively. The chemical shifts are reported in parts per million relative to TMS. The melting points (m.p.) were determined with an Electrothermal digital melting point apparatus and were uncorrected. The elemental analyses (EA) were performed by the Central Analysis Department of CNRS (Vernaison, France). The mass spectra (MS) were collected in electronic impact mode (70 eV) on a Varian 1200 MS/MS mass spectrometer coupled to a Varian 3800 gas chromatograph (GC). A Varian DB-5MS column (30 m; 0.25 mm; 0.25 μm) was used for the AME chromatography. The carrier gas was helium (1 mL/min). The injector (split ratio = 30) was held at 250 °C. The GC oven program was: 70 °C initial temperature, isothermal for 1.5 min, then 15 °C/min to 250 °C, and finally isothermal for 7 min. Thin-layer chromatography (TLC) was carried out on Merck 60 F254 precoated silica gel plates (0.25mm) using diethyl ether (100%) as eluent (*R*_F_ in Table 3). Spots were visualized successively with a UV lamp (λ=254 nm) and iodine vapors.

### Methyl 2-acetamidoacrylate synthesis (MAA; **2**)

Cs_2_CO_3_ (4.2 mmol) was added to **1** (1368 mg, 8.4 mmol) dissolved in MeOH (20 mL), and the mixture was stirred at room temperature for one hour. The methanolic solution was concentrated until dryness. Iodomethane (42 mmol; 2.7 mL), in controlled portions and undergoing constant stirring, was added over a two-hour period to the residue dissolved in DMF (30 mL). The reaction mixture was finally concentrated to dryness, and the crude residue, purified over a silica gel column using diethyl ether (Et_2_O 100%, *R*_F_ = 0.55) as eluent, gave 1199 mg of **2** as white needles, i.e. a yield of 99.7 %

*Methyl 2-acetamidoacrylate* (**2**). White solid, mp: 52-53°C; ^1^H-NMR (CDCl_3_): δ (ppm) 2.11 (s, 3 H, COCH_3_), 3.82 (s, 3 H, OCH_3_), 5.85 (s, 1H, CH_2_), 6.57 (s, 1H, CH_2_), 7.72 (s, 1H, NH); ^13^C-NMR (CDCl_3_): δ (ppm) 24.56 (CH_3_), 52.88 (CH_3_), 108.63 (CH_2_), 130.85 (C=CH_2_), 164.56 (CO_2_), 168.74 (CO); IR: ν (cm^-1^) 3364, 3155, 3057, 2963, 1813, 1708, 1678, 1629, 1512, 1428, 1369, 1327, 1246, 1208, 1172, 1038, 995, 977, 905, 856, 809; MS (m/z, EI): 143 (M^+^, 66), 111 (44), 101 (100), 71 (27), 43 (88), 42 (63); Anal. Calcd for C_6_H_9_NO_3_: C, 50.35; H, 6.34; N, 9.79; O, 33,53; Found: C, 50.41; H, 6.37; N, 9.39; O, 33,30.

### General procedure for methyl S-arylmercapturate (AME) synthesis: compounds ***3a-m***

Compounds **3b** to **3m** were synthesized using the parameters defined for **3a** in Table 2 entry 11 and in Table 3. Briefly, adequate thiophenol (1.4 mmol), Aliquat 336 (32 mg, 0.08 mmol) and finally powdered potassium carbonate (41 mg, 0.3 mmol) were added respectively to **2** (143 mg, 1 mmol) dissolved in toluene (30 mL, except if otherwise indicated in Table 3). The reaction mixture was stirred at room temperature and regularly monitored by thin layer chromatography using Et_2_O as eluent (R_F_, Table 3). The mixture filtered, the crude product, isolated after solvent removal, was subjected to silica gel column chromatography using Et_2_O as eluent. Yields are given in Table 3.

*2-Acetamido-3-p-tolylsulfanylpropionic acid methyl ester* (**3a**). White solid, mp: 84-85°C; ^1^H-NMR (CD_3_OD): δ (ppm) 1.88 (s, 3H, COCH_3_), 2.31 (s, 3H, CH_3_-Ar), 3.28-3.44 (m, 2H, SCH_2_), 3.56 (s, 3H, O-CH_3_), 4.60-4.63 (m, 1H, C**H**-CH_2_), 4.81-4.85 (m, 1H), 7.09-7.32 (m, 4H, ArH); ^13^C-NMR (CD_3_OD): δ (ppm) 20.1 (CH_3_), 21.4 (CH_3_), 36.2 (CH_2_), 51.8 (CH_3_), 52.7 (CH), 129.9 (CH_Ar_), 131.4 (CH_Ar_), 131.6 (C), 137.5 (C), 171.4 (CO), 172.2 (CO); IR: ν (cm^-1^) 3322, 3075, 2984, 2944, 2931, 2843, 1727, 1645, 1543, 1495, 1423, 1344, 1321, 1248, 1179, 1089, 1036, 803; MS (m/z, EI): 267 (M^+^, 15), 208 (80), 149 (30), 43 (100); Anal. Calcd for C_12_H_17_NO_3_S; C 58.40, H 6.41, N 5.24, O 17.95, S 11.99; Found: C 58.66, H 6.48, N 5.14, O 18.40, S 12.16.

*2-Acetamido-3-(4-ethylphenylsulfanyl)-propionic acid methyl ester* (**3b**). White solid, mp: 64-65°C; ^1^H-NMR (CD_3_OD): δ (ppm) 1.19 (t, ^3^*J* = 7.6 Hz, 3 H, CH_3_), 1.92 (s, 3 H, COCH_3_), 2.59 (q, ^3^*J* = 7.6 Hz, 2 H, CH_2_-CH_3_), 3.18 (dd, ^2^*J* = 14 Hz, ^3^*J* = 7.8 Hz, 1 H, SCH_2_), 3.34 (dd, ^2^*J* = 14 Hz, ^3^*J* = 4.8 Hz, 1 H, SCH_2_), 3.60 (s, 3 H, OCH_3_), 4.55 (dd, ^3^*J* = 7.8 Hz, ^3^*J* = 4.8 Hz, 1 H, CH_2_-CH), 4.84 (s, 1 H, NH), 7.13-7.16 (m, 2 H, 2-Ar-H), 7.32-7.35 (m,2 H, 2 Ar-H); ^13^C-NMR (CD_3_OD): δ (ppm) 16.2 (CH_3_), 22.4 (CH_3_), 29.5 (CH_2_), 37.3 (CH_2_), 53.0 (CH_3_), 53.8 (CH), 129.8 (CH_Ar_), 131.0 (C_Ar_), 132.7 (CH_Ar_), 145.0 (C_Ar_), 172.4 (CO), 173.2 (CO); IR: ν (cm^-1^) 3318, 3076, 2969, 2946, 2934, 1723, 1637, 1546, 1496, 1438, 1422, 1347, 1321, 1248, 1219, 1180, 1091, 1036, 1017, 814, 696; MS (m/z, EI): 281 (M^+^, 26), 222 (100), 207 (10), 190 (6), 163 (53), 151 (55), 136 (78), 123 (29), 105 (25), 88 (52), 77 (60), 59 (51); Anal. Calcd for C_14_H_19_NO_3_S; C 59.76, H 6.81, N 4.98, O 17.06, S 11.40; Found: C 59.82, H 6.79, N 4.90, O 17.35, S 11.46.

*2-Acetamido-3-o-tolylsulfanylpropionic acid methyl ester* (**3c**). White solid, mp: 66-67°C; ^1^H-NMR (CD_3_OD): δ (ppm) 1.95 (s, 3 H, COCH_3_), 2.38 (s, 3 H, CH_3_-Ar), 3.21 (dd, ^2^*J* = 14.0 Hz, ^3^*J* = 8.0 Hz, 1 H, SCH_2_), 3.39 (dd, ^2^*J* = 14.0 Hz, ^3^*J* = 4.9 Hz, 1 H, SCH_2_), 3.64 (s, 3 H, OCH_3_), 4.58 (dd, ^3^*J* = 8.0 Hz, ^3^*J* = 4.9 Hz, 1 H, C**H**-CH_2_), 7.12 – 7.22 (m, 3 H, Ar-H), 7.41 – 7.43 (m, 1 H, Ar-H); ^13^C-NMR (CD_3_OD): δ (ppm) 19.6 (CH_3_), 21.2 (CH_3_), 34.8 (CH_2_), 52.0 (CH_3_), 52.7 (CH), 126.9 (CH_Ar_), 127.4 (CH_Ar_), 130.5 (CH_Ar_), 130.7 (CH_Ar_), 134.1 (C_Ar_), 139.1 (C_Ar_), 171.3 (CO), 172.2 (CO); IR: ν (cm^-1^) 3311, 3069, 2952, 2940, 2838, 1736, 1650, 1542, 1432, 1335, 1223, 1168, 1036, 744; MS (m/z, EI): 267 (M^+^, 13), 208 (100), 149 (56), 137 (44), 134 (61), 91 (18), 43 (26); Anal. Calcd for C_12_H_17_NO_3_S; C 58.40, H 6.41, N 5.24, O 17.95, S 11.99; Found: C 58.28, H 6.45, N 5.08, O 18.19, S 12.19.

*2-Acetamido-3-(2-ethylphenylsulfanyl)propionic acid methyl ester* (**3d**). White solid, mp: 55-56°C; ^1^H-NMR (CD_3_OD): δ (ppm) 1,18 (t, ^3^*J* = 7.5 Hz, 3H, C**H**_3_-CH_2_), 1.94 (s, 3H, COCH_3_), 2.78 (q, ^3^*J* = 7.5 Hz, 2H, C**H**2-CH_3_), 3.19 (dd, ^2^*J* = 13.9 Hz, ^3^*J* = 8.1 Hz, 1 H, SCH_2_), 3.39 (dd, ^2^*J* = 13.9 Hz, ^3^*J* = 5,0 Hz, 1 H, SCH_2_), 3.62 (s, 3H, CH_3_-Ar), 4.55 (dd, ^3^*J* = 8.1 Hz, ^3^*J* = 5.0 Hz, 1 H, C**H**-CH_2_), 7.15 – 7.21 (m, 3 H, Ar-H), 7.41 – 7.44 (m, 1 H, Ar-H); ^13^C-NMR (CD_3_OD): δ (ppm) 15.7 (CH_3_), 22.4 (CH_3_), 28.2 (CH_2_), 36.5 (CH_2_), 53.0 (CH_3_), 53.7 (CH), 127.8 (CH_Ar_), 128.4 (CH_Ar_), 130.1 (CH_Ar_), 131.9 (CH_Ar_), 134.6 (C_Ar_), 146.3 (C_Ar_), 172.5 (CO), 173.3 (CO); IR: ν (cm^-1^) 3280, 3058, 2964, 2872, 1747, 1655, 1541, 1437, 1373, 1214, 1128, 1027, 752; MS (m/z, EI): 281 (M^+^, 42), 222 (88), 151 (30), 148 (100), 144 (25), 136 (45), 91 (12), 43 (21); Anal. Calcd for C_14_H_19_NO_3_S; C 59.76, H 6.81, N 4.98, O 17.06, S 11.40; Found: C 59.39, H 6.76, N 4.88, O 17.46, S 11.74.

2*-Acetamido-3-(3,5-dimethylphenylsulfanyl)propionic acid methyl ester* (**3e**). White solid, mp: 73-74°C; ^1^H-NMR (CD_3_OD): δ (ppm) 1.92 (s, 3 H, COCH_3_), 2.25 (s, 6 H, 2 CH_3_-Ar), 3.18 (dd, ^2^*J* = 14.2 Hz, ^3^*J* = 7.6 Hz, 1 H, SCH_2_), 3.35 (dd, ^2^*J* = 14.2 Hz, ^3^*J* = 4.8 Hz, 1 H, SCH_2_), 3.62 (s, 3 H, OCH_3_), 4.57 (dd, ^3^*J* = 7.6 Hz, ^3^*J* = 4.8 Hz, 1 H, CH_2_-CH), 4.84 (s, 1H, NH), 6.85 (m, 1 H, 1 Ar-H), 7.01 (m, 2 H, 2 Ar-H); ^13^C-NMR (CD_3_OD): δ (ppm) 21.4 (2 x CH_3_), 22.4 (CH_3_), 34.6 (CH_2_), 53.0 (CH_3_), 52.4 (CH), 129.3 (CH_Ar_), 131.1 (CH_Ar_), 131.5 (C_Ar_), 134.2 (C_Ar_), 170.1 (CO), 171.1 (CO); IR: ν (cm^-1^) 3269, 3072, 2953, 2914, 2856, 1757, 1733, 1652, 1549, 1430, 1376,1249, 1216, 1125, 1031, 841; MS (m/z, EI): 281 (M^+^, 25), 222 (45), 189 (8), 163 (100), 151 (42), 135 (19), 122 (14), 105 (51), 91 (36), 77 (34), 59 (35); Anal. Calcd for C_14_H_19_NO_3_S; C 59.76, H 6.81, N 4.98, O 17.06, S 11.40; Found: C 59.72, H 6.96, N 4.81, O 16.94, S 11.42.

*2-Acetamido-3-(3,4-dimethylphenylsulfanyl)propionic acid methyl ester* (**3f**). White solid, mp: 84-85°C; ^1^H-NMR (CD_3_OD): δ (ppm) 1.92 (s, 3 H, COCH_3_), 2.20 (s, 3 H, CH_3_-Ar(4)), 2.21 (s, 3 H, CH_3_-Ar (3)), 3.14 (dd, ^2^*J* = 14.0 Hz, ^3^*J* = 8.0 Hz, 1 H, SCH_2_), 3.31 (dd, ^2^*J* = 14.0 Hz, ^3^*J* = 4.8 Hz, 1 H, SCH_2_), 3.61 (s, 3 H, OCH_3_), 4.53 (dd, ^3^*J* = 8.0 Hz, ^3^*J* = 4.8 Hz, 1 H, CH_2_-CH), 4.84 (s, 1H, NH), 7.05 (m, 1 H, Ar-H), 7.13 (m, 1 H, Ar-H), 7.19 (m, 1 H, Ar-H); ^13^C-NMR (CD_3_OD): δ (ppm) 19.5 (CH_3_), 19.9 (CH_3_), 22.4 (CH_3_),37.3 (CH_2_), 52.9 (CH_3_), 53.8 (CH_3_), 130.2 (CH_Ar_), 131.4 (CH_Ar_), 132.6 (C_Ar_), 133.7 (CH_Ar_), 137.2 (C_Ar_), 138.7 (C_Ar_), 172.5 (CO), 173.2 (CO); IR: ν (cm^-1^) 3284, 3061, 3015, 2951, 2928, 2867, 1747, 1659, 1539, 1488, 1436, 1372, 1213, 1129, 1022, 814; MS (m/z, EI): 281 (M^+^, 18), 222 (65), 191 (7), 163 (100), 151 (55), 137 (16), 105 (53), 91 (38), 77 (49), 59 (28); Anal. Calcd for C_14_H_19_NO_3_S; C 59.76, H 6.81, N 4.98, O 17.06, S 11.40; Found: C 59.87, H 6.98, N 4.93, O 16.84, S 11.19.

*2-Acetamido-3-(2,4-dimethylphenylsulfanyl)propionic acid methyl ester* (**3g**). White solid, mp: 84-85°C; ^1^H-NMR (CD_3_OD): δ (ppm) 1.95 (s, 3 H, COCH_3_), 2.27 (s, 3 H, CH_3_-Ar (4)), 2.36 (s, 3 H, CH_3_-Ar (2)), 3.13 (dd, ^2^*J* = 14.0 Hz, ^3^*J* = 8.0 Hz, 1 H, SCH_2_), 3.30 (dd, ^2^*J* = 14.0 Hz, ^3^*J* = 5.0 Hz, 1 H, SCH_2_), 3.62 (s, 3 H, OCH_3_), 4.52 (dd, ^3^*J* = 8.0 Hz, ^3^*J* = 5.0 Hz, 1 H, CH_2_-CH), 4.86 (s, 1H, NH), 6.98 (m, 1 H, Ar-H), 7.04 (m, 1 H, Ar-H), 7.31 (m, 1 H, Ar-H); ^13^C-NMR (CD_3_OD): δ (ppm) 20.8 (CH_3_), 21.2 (CH_3_), 22.4 (CH_3_),36.5 (CH_2_), 53.0 (CH_3_), 53.7 (CH), 128.5 (CH_Ar_), 131.3 (C_Ar_), 132.4 (CH_Ar_), 132.9 (CH_Ar_), 138.6 (C_Ar_), 140.7 (C_Ar_), 172.5 (CO), 173.2 (CO); IR: ν (cm^-1^) 3293, 3063, 2959, 2916, 1749, 1652, 1546, 1435, 1411, 1376, 1292, 1258, 1218, 1170, 1127, 821; MS (m/z, EI): 281 (M^+^, 48), 222 (91), 207 (11), 190 (10), 163 (70), 151 (63), 138 (29), 122 (18), 105 (100), 91 (52), 77 (74), 59 (54); Anal. Calcd for C_14_H_19_NO_3_S; C 59.76, H 6.81, N 4.98, O 17.06, S 11.40; Found: C 59.76, H 6.86, N 4.82, O 17.40, S 11.38.

*2-Acetamido-3-(2,5-dimethylphenylsulfanyl)propionic acid methyl ester* (**3h**). White solid, mp: 84-85°C; ^1^H-NMR (CD_3_OD): δ (ppm) 1.95 (s, 3 H, COCH_3_), 2.29 (s, 3 H, CH_3_-Ar (5)), 2.33 (s, 3 H, CH_3_-Ar (2)), 3.18 (dd, ^2^*J* = 14.0 Hz, ^3^*J* = 8.0 Hz, 1 H, SCH_2_), 3.35 (dd, ^2^*J* = 14,0 Hz, ^3^*J* = 4.8 Hz, 1 H, SCH_2_), 3.63 (s, 3 H, OCH_3_), 4.57 (dd, ^3^*J* = 8.0 Hz, ^3^*J* = 4.8 Hz, 1 H, CH_2_-CH), 4.86 (s, 1H, NH), 6.95 (d, ^3^*J* = 7.8 Hz, 1 H, 1 Ar-H), 7.07 (d, ^3^*J* = 7.8 Hz, 1 H, 1 Ar-H), 7.24 (s, 1 H, 1 Ar-H); ^13^C-NMR (CD_3_OD): δ (ppm) 20.3 (CH_3_), 21.1 (CH_3_), 22.4 (CH_3_), 35.9 (CH_2_), 53.0 (CH_3_), 53.7 (CH), 129.0 (CH_Ar_), 131.4 (CH_Ar_), 132.2 (CH_Ar_), 134.7 (C_Ar_), 137.0 (C_Ar_), 137.4 (C_Ar_), 172.4 (CO), 173.3 (CO); IR: ν (cm^-1^) 3319, 3004, 2952, 2915, 1769, 1651, 1530, 1489, 1432, 1379, 1251, 1216, 1189, 1171, 1029, 976, 800; MS (m/z, EI): 281 (M^+^, 25), 222 (73), 190 (10), 163 (100), 149 (98), 135 (25), 122 (15), 105 (84), 91 (58), 77 (78), 59 (58); Anal. Calcd for C_14_H_19_NO_3_S; C 59.76, H 6.81, N 4.98, O 17.06, S 11.40; Found: C 59.91, H 6.84, N 4.95, O 17.41, S 11.52.

*2-Acetamido-3-phenylsulfanylpropionic acid methyl ester* (**3i**). White solid, mp: 64-65°C; ^1^H-NMR (CD_3_OD): δ (ppm) 1.96 (s, 3 H, COCH_3_), 3.23 (dd, ^2^*J* = 14.0 Hz, ^3^*J* = 8.0 Hz, 1 H, SCH_2_), 3.40 (dd, ^2^*J* = 14,0 Hz, ^3^*J* = 5.2 Hz, 1 H, SCH_2_), 3.62 (s, 3 H, OCH_3_), 4.68 (dd, ^3^*J* = 8.0 Hz, ^3^*J* = 5.2 Hz, 1 H, CH_2_-CH), 4.85 (s, 1H, NH), 7.20-7.24 (m, 1 H, 1 Ar-H), 7.28-7.32 (m, 2 H, 2 Ar-H), 7.39-7.43 (m, 2 H, 2 Ar-H); ^13^C-NMR (CD_3_OD): δ (ppm) 22.4 (CH_3_), 36.6 (CH_2_), 53.0 (CH_3_), 53.8 (CH), 128.1 (CH_Ar_), 130.3 (CH_Ar_), 131.8 (CH_Ar_), 136.3 (C_Ar_), 172.4 (CO), 173.3 (CO); IR: ν (cm^-1^) 3283, 3058, 2952, 2849, 1747, 1653, 1539, 1482, 1438, 1373, 1306, 1214, 1177, 1128, 1025, 743; MS (m/z, EI): 253 (M^+^,10), 194 (84), 163 (14), 135 (100), 123 (73), 109 (45), 88 (42), 77 (35), 65 (47); Anal. Calcd for C_12_H_15_NO_3_S; C 56.90, H 5.97, N 5.53, O 18.95, S 12.66; Found: C, 57.35; H, 5.91; N, 5.32; O, 18.75; S, 12.66.

*2-Acetamido-3-(4-chlorophenylsulfanyl)propionic acid methyl ester* (**3j**). White solid, mp: 106-107°C; ^1^H-NMR (DMSO-d_6_): δ (ppm) 1.83 (s, 3 H, COCH_3_), 3.19 (dd, ^2^*J* = 13.8 Hz, ^3^*J* = 8.0 Hz, 1 H, SCH_2_), 3.32 (dd, ^2^*J* = 13.8 Hz, ^3^*J* = 5.4 Hz, 1 H, SCH_2_), 3.57 (s, 3 H, OCH_3_), 4.40 (dd, ^3^*J* = 8.0 Hz, ^3^*J* = 8.0 Hz, ^3^*J* = 5.4 Hz, 1 H, C**H**-CH_2_), 7.36 (s, 4 H, Ar-H), 8.50 (d, ^3^*J* = 8.0 Hz, 1H, NH); ^13^C-NMR (DMSO-d_6_): δ (ppm) 22.4 (CH_3_), 34.6 (CH_2_), 51.8 (CH_3_), 52.4 (CH), 129.3 (CH_Ar_), 131.1 (CH_Ar_), 131.5 (C_Ar_), 134.2 (C_Ar_), 170.1 (CO), 171.1 (CO); IR: ν (cm^-1^) 3308, 3075, 2951, 2930, 1730, 1643, 1546, 1478, 1425, 1369, 1349, 1247, 1219, 1192, 1177, 1099, 1037, 1011, 808; MS (m/z, EI): 287 (M^+^,11), 230 (41), 228 (100), 197 (10), 169 (50), 157 (22), 143 (15), 108 (12), 88 (29); Anal. Calcd for C_12_H_14_ClNO_3_S; C 50.09, H 4.90, Cl 12.32, N 4.87, O 16.68, S 11.14; Found: C 50.80, H 5.23, Cl 11.93, N 4.68, O 16.20, S 10.54.

*2-Acetamido-3-(4-bromophenylsulfanyl)propionic acid methyl ester* (**3k**). White solid, mp: 121-122°C; ^1^H-NMR (CDCl_3_): δ (ppm) 1.80 (s, 3H, COCH_3_), 3.21 (dd, ^2^*J* = 14.2 Hz, ^3^*J* = 4.7 Hz, 1 H, SCH_2_), 3.35 (dd, ^2^*J* = 14.2 Hz, ^3^*J* = 4.7 Hz, 1 H, SCH_2_), 3.49 (s, 3H, OCH_3_), 4.73 (m, 1 H, C**H**-CH_2_), 6.16 (s, 1H, NH), 7,14-7,16 (m, 2 H, Ar-H), 7.29 – 7.31 (m, 2 H, Ar-H); ^13^C-NMR (CDCl_3_): δ (ppm) 22.9 (CH_3_), 36.5 (CH_2_), 52.2 (OCH_3_), 52.5 (CH), 121.0 (C_Ar_), 132.1 (CH_Ar_), 132.3 (CH_Ar_), 134.0 (C_Ar_), 169.6 (CO), 170.6 (CO); IR: ν (cm^-1^) 3307, 3047, 2950, 1729, 1641, 1546, 1474, 1425, 1350, 1324, 1245, 1176, 1092, 1039, 1006, 806; MS (m/z, EI): 333 (18), 331 (M^+^, 17), 274 (99), 272 (100), 215 (10), 213 (20), 203 (13), 201 (13), 189 (11), 187 (11), 144 (18), 134 (53), 122 (67), 108 (50), 88 (74), 75 (25), 60 (60); Anal. Calcd for C_12_H_14_BrNO_3_S: C, 43.38; H, 4.25; Br, 24.05; N, 4.22; O, 14.45; S, 9.65. Found: C,44.56, H, 4.41, Br, 23.59, N, 4.04, O, 14.38, S, 9.29.

*2-Acetamido-3-(4-methoxyphenylsulfanyl)propionic acid methyl ester* (**3l**). Colorless oil; ^1^H-NMR (CDCl_3_): δ (ppm) 1.89 (s, 3H, COCH_3_), 3.22 (dd, ^2^*J* = 14.2 Hz, ^3^*J* = 5.0 Hz, 1 H, SCH_2_), 3.32 (dd, ^2^*J* = 14.2 Hz, ^3^*J* = 4.7 Hz, 1 H, SCH_2_), 3.54 (s, 3H, OCH_3_), 3.76 (s, 3 H, Ar-OCH_3_), 4.77 (m, 1 H, C**H**-CH_2_), 6.81 – 6.83 (m, 2 H, Ar-H), 7.35 – 7.37 (m, 2 H, Ar-H); ^13^C-NMR (CDCl_3_): δ (ppm) 22.9 (CH_3_), 38.0 (CH_2_), 52.2 (OCH_3_), 52.3 (CH), 55.3 (OCH_3_), 114.7 (CH_Ar_), 124.7 (C_Ar_), 134.2 (CH_Ar_), 159.4 (C_Ar_), 169.7 (CO), 170.8 (CO); IR: ν (cm^-1^) 3289, 3065, 2953, 2838, 1746, 1656, 1592, 1541, 1494, 1437, 1373, 1286, 1246, 1175, 1029, 827; MS (m/z, EI): 283 (47), 224 (100), 165 (27), 153 (22), 144 (25), 165 (27), 109 (9), 84 (7); Anal. Calcd for C_13_H_17_NO_4_S: C, 55.11; H, 6.05; N, 4.94; O, 22.59; S, 11.32. Found: C, 55.27; H, 6.32; N, 4.87; O, 22.79; S, 11.30.

*2-Acetamido-3-pentachlorophenylsulfanylpropionic acid methyl ester* (**3m**). White solid, mp: 145-146°C; ^1^H-NMR (DMSO-d_6_): δ (ppm) 11.75 (s, 3 H, COCH_3_), 3.17 (dd, ^2^*J* = 14.0 Hz, ^3^*J* = 8.8 Hz, 1 H, SCH_2_), 3.40 (dd, ^2^*J* = 14.0 Hz, ^3^*J* = 4.8 Hz, 1 H, SCH_2_), 3.57 (s, 3 H, OCH_3_), 4.36 (ddd, ^3^*J* = 8.8 Hz, ^3^*J* = 8.0 Hz, ^3^*J* = 4.8 Hz, 1 H, C**H**-CH_2_), 8.36 (d, ^3^*J* = 8.0 Hz, 1H, NH); ^13^C-NMR (DMSO-d_6_): δ (ppm) 22.4 (CH_3_), 36.0 (CH_2_), 52.5 (CH), 52.8 (CH_3_), 131.4 (C_Ar_), 133.5 (C_Ar_), 134.4 (C_Ar_), 138.2 (C_Ar_), 170.0 (CO), 170.8 (CO); IR: ν (cm^-1^) 3293, 2956, 1758, 1651, 1532, 1435, 1378, 1334, 1305, 1259, 1215, 1190, 1170, 1130, 688; MS (m/z, EI): 425 (M^+^, 5), 366 (100), 324 (13), 246 (18), 167 (12), 144 (37), 117 (48), 102 (18), 88 (90), 69 (34); Anal. Calcd for C_12_H_10_Cl_5_NO_3_S; C 33.87, H 2.37, Cl 41.66, N 3.29, O 11.28, S 7.54; Found: C 34.24, H 2.58, Cl 42.01, N 2.97, O 10.75, S 6.96.
